# Challenges in precision medicine in pancreatic cancer: A focus in cancer stem cells and microbiota

**DOI:** 10.3389/fonc.2022.995357

**Published:** 2022-12-01

**Authors:** Catalina M. Perelló-Reus, Teresa Rubio-Tomás, Eugenia Cisneros-Barroso, Lesly Ibargüen-González, Juan José Segura-Sampedro, Rafael Morales-Soriano, Carles Barceló

**Affiliations:** ^1^ Translational Pancreatic Cancer Oncogenesis Group, Health Research Institute of the Balearic Islands (IdISBa), Hospital Universitari Son Espases (HUSE), Palma de Mallorca, Spain; ^2^ School of Medicine, University of Crete, Herakleion, Greece; ^3^ Internal Medicine Department, Son Llàtzer University Hospital, Palma de Mallorca, Spain; ^4^ Advanced Oncological Surgery, Health Research Institute of the Balearic Islands (IdISBa), Palma de Mallorca, Spain; ^5^ General and Digestive Surgery Unit, Hospital Universitari Son Espases, School of Medicine, Balearic Islands Health Research Institute, University of Balearic Islands, Palma de Mallorca, Spain

**Keywords:** precision medicine, PDAC, cancer stem cell, niche factors, microbiota, early diagnosis, therapy resistance, organoid co-culture

## Abstract

Pancreatic cancer adenocarcinoma (PDAC) is a lethal disease, with the lowest 5-years survival rate of all cancers due to late diagnosis. Despite the advance and success of precision oncology in gastrointestinal cancers, the frequency of molecular-informed therapy decisions in PDAC is currently neglectable. The reasons for this dismal situation are mainly the absence of effective early diagnostic biomarkers and therapy resistance. PDAC cancer stem cells (PDAC-SC), which are regarded as essential for tumor initiation, relapse and drug resistance, are highly dependent on their niche *i.e.* microanatomical structures of the tumor microenvironment. There is an altered microbiome in PDAC patients embedded within the highly desmoplastic tumor microenvironment, which is known to determine therapeutic responses and affecting survival in PDAC patients. We consider that understanding the communication network that exists between the microbiome and the PDAC-SC niche by co-culture of patient-derived organoids (PDOs) with TME microbiota would recapitulate the complexity of PDAC paving the way towards a precision oncology treatment-response prediction.

## Introduction

Precision medicine (PM) involves the customization of healthcare for a specific individual on the basis of biomarker measurements obtained at the individual and population levels (1). Remarkably, in the last years the management of cancers of the gastrointestinal system is moving towards a precision medicine paradigm (2) in which biomarkers for precision medicine are a topic of intense research (3, 4).

In the last decade it has been established the central role of cancer stem cells (CSC)- i.e. the subpopulation of cancer cells capable of self-renewing and producing progeny- in the progression, treatment resistance and metastasis of gastrointestinal cancer (5–7). CSCs depend on their niches, which are anatomically distinct regions within the tumor microenvironment (TME). These niches maintain the principal properties of CSCs, preserve their phenotypic plasticity, protect them from the immune system and facilitate their metastatic potential (8–10). Interestingly, biomarkers related to CSCs and its niche/TME have been found to be among the most accurate in prediction of disease progression and, specially, disease recurrence (11–13) and also to develop tailored therapies that optimize patient’s opportunities to cure (14)

A variety of tumors contain bacteria what suggests that the microbiome could play a role in the TME (15). In fact, the microbiome is proposed to have an active involvement in the pathogenesis and treatment responses. This is in line with the view that tumors should be treated as biosystems instead of only a set of transformed epithelial cells (16). Specifically, microbiota-related biomarkers have recently been posed both as predictors of disease progression and treatment response (17), and as relevant targets of anti-cancer therapies in many malignancies (18). Thus, studying the interplay between cancer stem cells and intratumoral microbiota seems to be a promising strategy in the development of new biomarkers for a cancer precision medicine.

## Challenges in PDAC personalized treatment

Pancreatic ductal adenocarcinoma (PDAC) is a lethal disease, with the lowest 5-years survival rate of all cancers (18)Although PDAC presents low frequency (incidence of 8–12 cases per 100 000 people per year, and a 1·3% lifetime risk for the disease) will be the second cancer-related death reason in 2040 in the USA (18). Despite the advance and success of precision oncology in gastrointestinal cancers, the frequency of molecular-informed therapy decisions in PDAC is currently neglectable (19)Therefore, understanding the pathogenesis of this lethal disease is urgently needed to stratify patients and to develop personalized novel therapeutic approaches for it.

Current therapies rely on conventional polychemotherapies with poor outcomes and molecular-informed targeted therapy opportunities only exist in a tiny minority of patients (19). This clearly demonstrates that the tremendous potential of genetically guided precision oncology used in other GI malignancies (2, 20), in PDAC meets important limitations. For that reason, there is the need to expand the knowledge about PDAC biology in order to decipher other targetable mechanism such as tumor microenvironment and cellular plasticity (5, 21)

Cellular plasticity is the ability of tumor cells to adapt to changing conditions by acquiring different molecular and phenotypic identities and, thereby, plasticity programs are key regulators of acquired treatment resistance (22).For this purpose, one of the most critical questions in both cancer research and clinic is how PDAC is maintained and expanded after it has emerged. Cancer stem cells (CSCs), and particularly PDAC stem cells (PDAC-SC) have been considered as a subpopulation of cancer cells capable of self-renewing and producing progeny cells that are critical for cancer growth (23, 24). This mechanism may underlie the maintenance of cancer and its resistance to conventional therapies.

According to the current model, CSCs are not a fixed cell population but a plastic one, i.e. the aforementioned characteristics can be acquired and lost dependent on environmental stimuli (25)Therefore, CSC are highly dependent on their niche, i.e. microanatomical structures of TME in which CSCs are maintained and protected from therapy (26, 27)

In this regard, in an unbiased approach, clonogenic capacity of PDAC-SC was shown to be fully defined by the microenvironment and not by tumor-cell-intrinsic-features (28) confirming a dichotomous role of stroma either promoting or inhibiting PDAC-SC tumorigenic capacity (29, 30)

We believe that the PDAC CSC (PDAC-SC) biology is strongly affected by the interplay between the genetic alteration and the tumor microenvironment, particularly the microbiome. Unraveling the link between microbiota and cancer stem cells technologies will provide insights into the pathology of cancers of the gastrointestinal system, as well as promote the translation of these findings to the clinics towards personalized medicine.

### Late diagnostic

Given the dismal prognosis of PDAC patients, early and differential diagnosis of severe pancreatic cancers is essential and challenging for patients with PDAC and constitutes an unmet clinical problem (18). Symptoms are unspecific and often emerge only during late disease stages, at which point, tumors can be either locally non-resectable or present as metastatic disease. At present, PDAC is diagnosed using imaging tests and currently, despite other promising circulating biomarkers have been described (31) the sole FDA-approved biomarker for PDAC is serum CA19-9, mostly used for disease monitoring rather than screening, due to inherent limits of sensitivity and specificity: CA19-9 levels can be elevated in several conditions unrelated to pancreatic cancer, while subjects lacking the Lewis-A antigen do not produce CA19-9 at all (32). Thus, the outcome of PDAC patients could improve with sensitive and affordable tests that would permit early detection of the disease.

A plethora of studies have shown that microbiota most likely affects the malignant phenotype and prognosis of PDAC (33, 34)Therefore, microbiome signatures enable robust metagenomic classifiers for PDAC detection at high disease specificity and with potential towards cost-effective PDAC screening and monitoring. Interestingly, in a recent study (35), showed that faecal metagenomic classifiers had much better performance than saliva-based classifiers and could identify patients with PDAC with an AUC score of up to 0.84 based on a set of 27 microbial species, with consistent accuracy across early and late disease stages, increasing when combined with serum levels of CA19-9, indicating the potential for non-invasive, robust, and specific faecal microbiota-based early diagnosis for PDAC (35)

Many studies suggest that quiescent plastic CSCs are already present but resting/latent during early stages of disease development (26, 36). Importantly, early quiescent PDAC-SCs initiate KRAS mutant pancreatic lesions leading to PDAC in the context of pancreatitis (37, 38) a condition known to be heavily influenced by microbiome (33). Interestingly, circulating PDAC circulating tumor cells with stem-like characteristics could be used as an early PDAC biomarker (39).

### Therapy resistance

**Figure 1:**
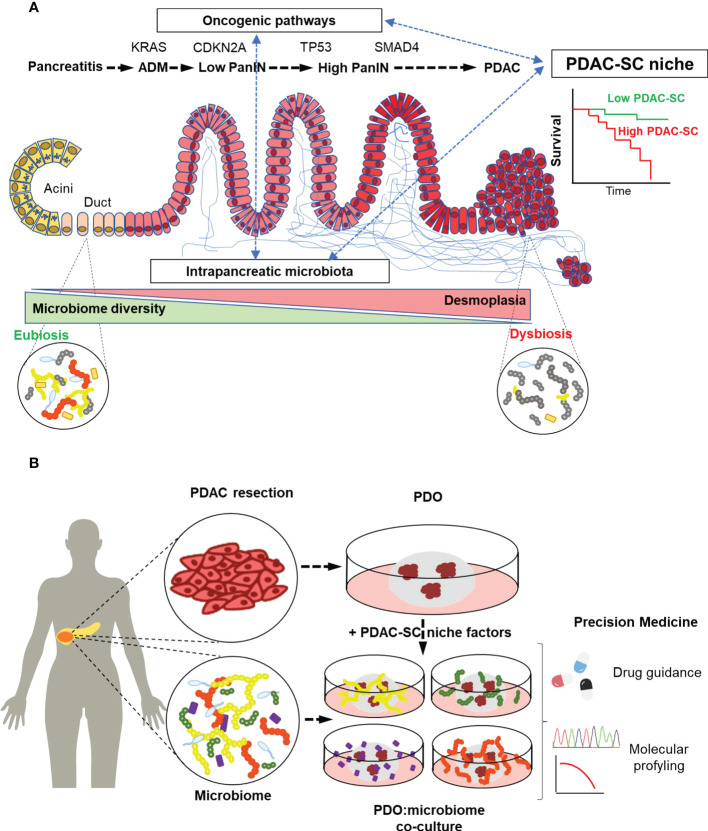
Intratumoral Microbiota may define PDAC stem cell niche, thereby constituting a diagnosis and prognosis biomarker. **(A)** In the development of PDAC, driver mutations accumulation is accompanied by an increasing desmoplastic reaction (blue lines) as a hallmark histopathological feature in PDAC stroma. Microbiota embedded in the desmoplastic stroma changes towards a dysbiotic low-diversity composition that might impact the PDAC stem cell niche by favoring tumor progression and resistance to chemotherapy. **(B)** Patient-derived organoids (PDOs) are generated mainly from PDAC resection containing PDAC cancer stem cells (PDAC-SC). PDOs can be co-cultured with the patient microbiota to recapitulate the PDAC-SC niche. This co-culture technique paves the way to the study of microbiome-focused precision medicine bench-to-beside approaches to overcome the lack of early diagnosis and therapy resistance in PDAC.

The accumulation of driver mutations is accompanied by histological changes that represent the different stages of PDAC development. Morphological evolution begins with the formation of precursor lesions, termed pancreatic intraepithelial neoplasia (PanIN), with increasing histological grades followed by progression to invasive adenocarcinoma (**Figure 1A**).

A histopathological hallmark of PDAC is a desmoplastic reaction to the tumor that is present in both primary and metastatic tumors (40). Pancreatic stellate cells, a myofibroblast-like type of cell in the pancreas are activated by cancer cells to produce high fibrosis surrounding the tumor (41).The resultant desmoplasia is known to be responsible for creating a mighty mechanical barrier around the tumor cells, preventing appropriate vascularization, and thus limiting exposure to chemotherapy and largely preventing immune cell infiltration (42)

Early research largely stemmed from the idea that the surrounding desmoplasia is tum or promoting but this view of its role is most probably an imperfect one (29). Therapeutic approaches to target stromal desmoplasia have classically focused on depleting the stromal constituents but results have been generally disappointing, owing to the multi-faceted nature of tumor stroma (43). Furthermore, TME composition is a cell-extrinsic factor that influences the transcriptional landscape. Depending on the mRNA expression two major tumor subtypes have been described: basal or classical (44, 45). Interestingly, basal subtype presents stem-like properties (46), which interestingly correlates with dismal prognosis (47) and poor gemcitabine response (48).

In this regard, the intestinal microbiome has recently gained increasing interest in the field of PDAC TME with studies suggesting a tumorigenic relevance of bacterial dysbiosis within the TME. Since the early evidence of bacteria presence in PDAC TME (16, 34) and despite substantial inter-individual variability of the gut flora, some studies concur in their findings, pointing at different bacterial species potentially involved in PDAC tumorigenesis thought their interaction with the desmoplastic stroma (49). The formation of a new desmoplastic niche that offers lower colonization resistance and provides nutrition in the form of increased glycan levels might favor the migration of specific bacteria (25, 50, 51). In turn, new resident bacteria might remodulate the TME to promote tumor development and progression by favoring a PDAC-SC niche refractive to chemotherapy by inducing EMT-dependent stemness state or metabolizing chemotherapeutic agents (25, 34, 51), processes that could even cooperate to enhance therapy resistance (52).

The most prominent, although not exclusive, microbes identified in pancreatic tissue samples and associated with PDAC TME are Gram-negative bacteria, more specifically from the phylum *Proteobacteria* (25, 50, 53). Among *Proteobacteria, Gammaproteobacteria* was associated with poor patient prognosis (34, 53). These bacteria express the enzyme cytidine deaminase which enables the metabolization of the chemotherapeutic drug gemcitabine (2′,2′-difluorodeoxycytidine), which is commonly used for treatment of PDAC patients in the adjuvant and palliative setting, into its inactive form (2′,2′-difluorodeoxyuridine) (40). This might synergize with a quiescent PDAC-SC subpopulation, able to evade chemotherapeutic anti-tumor therapies (54) which is a hallmark of plastic PDAC-SC and responsible for disease relapse years after successful surgical intervention or tumor free survival (55).

Importantly, a distinct tumor microbiome was shown to clearly discriminate long-term survivor (median survival: 9.66 years) from short-term survivor (median survival: 1.66 years) PDAC patients with a strong correlation between dismal prognosis and low diversity (56). Long-term-survivors (LTS) contain higher alpha-diversity, presenting also Gram positive classes such as *Clostridia* and *Actinomycetia*, and a LTS-specific intra-tumoral microbiome signature was described (56). Of note, elevated levels of single microbial species correlated with poor prognosis, making diversity analysis, even in the stools an attractive, cheap, and non-invasive method to predict prognosis (35, 57).

## Personalized medicine in PDAC: A holistic ex vivo co-culturing modeling to predict treatment response

To follow a personalized medicine approach, there is an urgent need to find a model that recapitulates the tumor characteristics and that could be generated in a useful time frame taking into account the time constraints of PDAC management. Current models show limitations. PDAC derived 2D cell lines fail in reproducing the polarity, microenvironment, cell metabolism and gene expression which can affect drug response prediction (58). Patient-derived xenografts (PDX) better reproduce the tumor and predict drug response but the use of PDX remains challenging due to time concerns and the lack of a human microenvironment.

Alternatively, organoids are a 3D model derived from CSC that can be generated in 2-4 weeks and maintain the histological and genetic features of the tumor of origin. In 2013, Huch et al. described for the first time the generation of pancreatic organoids (59). Several years later, the same model was used to generate PDAC organoids from mouse and human tissue defining the medium composition (60). This offers the possibility to design a personalized medicine approach by using primary PDAC patient material (tumor resection or fine needle biopsy) to generate patient-derived-organoids (PDOs) that can work as patient avatars to predict the therapy response. PDOs can be also generated from human induced pluripotent stem cells (61).

In that sense, different PDAC PDOs biobanks have been generated (30, 60, 62, 63). PDAC organoids recapitulate the mutation profile of the original tumor (62, 63) and have been demonstrated to be a valuable tool to test drug sensitivity (48, 62–64) that even allows the study of drug-induced vulnerabilities in tumor relapse that can be therapeutically exploited in a bench-to-bedside approach (19).

Nevertheless, PDOs present some limitations such as that the drug response can be modified by the transcriptional changes due to the bottleneck of medium composition. In fact, PDOs transcriptional landscape depends on culture conditions favoring certain subtypes (63). Furthermore, dependence on growth factors and medium composition can exert a selective pressure to select the organoids containing the driver mutations of PDAC (30).

The lack of physiological niche factors could be bypassed by the use of tumor-on-a-chip devices that reproduces the TME. This includes the incorporation of stromal cells (65, 66) what highlights the importance of the co-culture with other cell types in order to mimic the complexity of the tumor. This is particularly relevant in PDAC, a tumor characterized by its low cellularity. Organotypic co-culture models have been established in PDAC PDOs. In that sense, PDOs have been co-cultured with cancer associated fibroblasts (CAFs) (67–70) and infiltrating lymphocytes isolated from blood (67) Interestingly, in co-culture experiments, Lodestijn et al. demonstrated that the factors secreted by TME maintain populations of tumor cmells with clonogenic potential (28).This shows the importance of co-culture PDAC-SC with the stromal factors responsible for maintaining CSCs and/or promoting the dedifferentiation of non-CSC tumor cells.

Since tumor microbiome is clearly affecting PDAC oncogenesis (34, 53, 71–73) this could be considered another key TME element with an impact on PDAC drug screening. The addition of the purified tumor microbiome to the PDO models would add a layer of complexity to the *in vitro* modeling of this dismal prognosis disease, better reproducing the tumor characteristics anticipating a good predictive drug response tool. In particular, it could be interesting to study the effect of microbiome on PDAC-SC population. As far as we know, the co-culture of organoids and microbiome has not been done yet in PDAC but there are established protocols in intestinal models (74, 75). The microinjection of the bacteria into the lumen mimics the microbiome habitat (75). However, the manipulation of specific factors (e. g. oxygen and nutrient levels) to allow the co-culture of a diversity of bacteria with organoids remain to be established.

Co-culture experiments could be used to envisage the effect of tumor microbiota on CSCs. Furthermore, developing a model able to recapitulate the complexity of PDAC-SC niche (**Figure 1B**) paves the way towards a more accurate and physiological treatment-response prediction capacity of cultured PDOs.

## Molecular studies to uncover microbiome- stem cell niche crosstalk

As stated before, *in vitro* models aiming to recapitulate the complexity of PDAC-SC niche need to include the microbiome axis to fully define the complex crosstalk between stem cells and microbiota.

The controlled escalation of biological complexity on the host side as well as in the composition of microbiome-derived secreted factors or live bacterial communities enable the proof-of-concept of a complex interaction mechanism in a controlled and standardized environment. These models open the door to a new generation of molecular studies difficult to study *in vivo*.

Microbial communities in the gut are known to produce small molecules and metabolites that significantly contribute to host functions and homeostasis (76). This interplay has been extensively studied in the intestine using microbiome-organoid co-culture models. In this regard, Sodhi et al. (28) found that bacterial Lipopolysaccharide (LPS) activates Toll-like receptor 4 (TLR-4) and enhances cell differentiation of goblet cell lineages in colonic organoids but inhibits Lgr5+ colon stem cells (77, 78) Similarly, a recent study found that dietary raffinose is utilized by *Lactobacillus reuteri* to convert it to fructose which in turn engages glycolysis to fuel stem cell proliferation under stress conditions (79).

As stated before, some bacterial families are conducive to oncogenesis and progression, while others prevent tumor development and might aid innate and therapeutically induced anti-tumor immunity. However, studying microbiome effects on tumor-related immunity in *ex vivo* systems is challenging, normally forcing the use of *in vivo* models which makes it difficult to dissect direct effects of microbiota on immune cells. Again, the use of microbiome-organoid co-culture approaches could circumvent the difficulties. In this regard, a recent study developed a novel immune-enhanced tumor organoid system to study factors affecting Immune Checkpoint Blockade (ICB) response. Selective testing of bacterial-derived metabolites from species found in the immunomodulatory host-microbiome significantly increased ICB-induced apoptosis of tumor cells and altered immune cell receptor expression (80).

Organoid have been used extensively to model senescence and aging-related conditions (81). In this regard, a recent study (82) found that gut microbiota metabolite trimethylamine N-oxide induces aging-associated senescent phenotype in midbrain organoids. Also, with these models, stem cell DNA damage associated to microbiota could be studied. Microbial co-culture with gastric organoids uncovered the mechanism by which *Helicobacter pylori* favours the accumulation of DNA damage promoting gastric cancer. In this regard (83), reported that DNA damage by *H. pylori* occurs in an ALPK1/TIFA/NF-KB-dependent manner in S-phase cells and importantly, the *H. pylori* LPS precursor (β-ADP-heptose) was sufficient to induce this damage.

Similar approaches could indeed be used to isolate individual microbiome-induced factors that alter PDAC-SC niche with the intrinsic limitation of the complexity of PDAC TME defined above. Although a co-culture of microbiome and escalation of biological complexity on the host side is possible, certain hallmarks of PDAC such as the strong desmoplastic reaction and the organoid bias towards a classical subtype (84) would be challenging to fully recapitulate PDAC complexity at the experimental level.

## Discussion and future prospects: Towards a microbiome-targeted precision medicine

Current research in the personalized medicine field promises new hope for developing new tools for early diagnosis and for improving treatment of this deadly disease. Along these lines, our ever-expanding understanding of PDAC-SC and the interplay between intratumoral microbiome and oncogenes in all aspects of PDAC is promising. We now know that PDAC-SC play a fundamental role in the initiation and development of PDAC, and these cells are largely responsible for the aggressive, chemoresistant and metastatic nature of this cancer (26, 37, 85) (32, 43, 74). They are known to be dependent on niche factors (28–30). Thus, understanding the communication network that exists within the TME, including the PDAC-SC niche, are not only important for understanding PDAC pathogenesis, but may also be relevant at the level of resistance to conventional therapies and cellular plasticity.

As outlined above, evidence supporting a tumor-promoting role of an altered host microbiome at different sites is accumulating (34, 53, 71). This altered diversity may be a consequence of tumorigenesis, as the evolution of an inflammatory tumor microenvironment might promote bacterial translocation from the gut into the pancreas (25, 34, 56). Considering all the above evidence it is reasonable to speculate that the interplay between the intratumoral microbiota and oncogenic mutations promotes a specific PDAC-SC niche thereby impacting the tumor progression, chemoresistance and patient prognosis.

This interplay seems to be important in other gastrointestinal malignancies such as gastric cancer (86), esophageal cancer (87, 88). In this line, 26 microbial markers were proposed as early detection biomarkers to discriminate adenoma from colorectal cancer (89), and 30 microbial markers were identified and validated as diagnosis biomarkers in cohorts of individuals with early hepatocellular carcinoma and healthy controls (90).

In the case of PDAC, an improvement in treatment response due to the modulation of the patient’s microbiome is already proposed in preclinical studies. Some even demonstrate a potential modulation of PDAC intratumoral microbiota with specific antibiotics overcoming gemcitabine and immunotherapy resistance in mouse models (57). In this regard, clinical trials focusing on compiling 16S rRNA profiles of PDAC patient samples and modulating microbiota are on the rise (based on http://clinicaltrials.gov/)

There is mounting evidence that patient microbiome composition can be used as a biomarker for disease progression as well as a druggable target to increase therapeutic efficacy of PDAC treatment. Therefore microbiome modulating strategies targeting the microbiome-dependent PDAC-SC niche would increase therapeutic responses and survival of PDAC patients paving the way towards the cure of this deadly disease.

## Author contributions

Conceptualization and methodology: CMP-R and CB. Writing—original draft preparation, CMP-R and CB. Writing—review and editing, CMP-R, TR-T, LI-G, EC-B, JS-S, RM-S and CB. Supervision: CB. Project administration and funding acquisition: CB. All authors contributed to the article and approved the submitted version.

## Funding

This work was funded by the Instituto de Salud Carlos III (ISCIII) and Programa de Investigación en Salut—ISCIII (PI20/01267) cofunded by the European Union and through the Programa Miguel Servet (MS19/00100) cofunded by the European Regional Development Fund/European Social Fund, "A way to make Europe"/"Investing in your future" (CB); the FOLIUM fellowship program (FOLIUM 19/01), Impost turisme sostenible/Govern de les Illes Balears (CMP-R) and TECH fellowship program, Impost turisme sostenible/Govern de les Illes Balears (TECH19/03) (LI-G).

## Conflict of interest

The authors declare that the research was conducted in the absence of any commercial or financial relationships that could be construed as a potential conflict of interest.

## Publisher’s note

All claims expressed in this article are solely those of the authors and do not necessarily represent those of their affiliated organizations, or those of the publisher, the editors and the reviewers. Any product that may be evaluated in this article, or claim that may be made by its manufacturer, is not guaranteed or endorsed by the publisher.

## References

[1] Tebani A Afonso C Marret S Bekri S . Omics-based strategies in precision medicine: Toward a paradigm shift in inborn errors of metabolism investigations. Int J Mol Sci (2016) 17(9):1555. doi: 10.3390/ijms17091555 27649151 PMC5037827

[2] Matsuoka T Yashiro M . Precision medicine for gastrointestinal cancer: Recent progress and future perspective. World J Gastrointest Oncol (2020) 12(1):1–20. doi: 10.4251/wjgo.v12.i1.1 31966910 PMC6960076

[3] Kristensen LS Jakobsen T Hager H Kjems J . The emerging roles of circRNAs in cancer and oncology. Nat Rev Clin Oncol (2022) 19(3):188–206. doi: 10.1038/s41571-021-00585-y 34912049

[4] Sankar K Ye JC Li Z Zheng L Song W Hu-Lieskovan S . The role of biomarkers in personalized immunotherapy. Biomark Res (2022) 10(1):32. doi: 10.1186/s40364-022-00378-0 35585623 PMC9118650

[5] Yang H Jiang Q . A multi-omics-based investigation of the immunological and prognostic impact of necroptosis-related genes in patients with hepatocellular carcinoma. J Clin Lab Anal (2022) 36(4):e24346. doi: 10.1002/jcla.24346 35293027 PMC8993599

[6] Nguyen LV Vanner R Dirks P Eaves CJ . Cancer stem cells: an evolving concept. Nat Rev Cancer (2012) 12(2):133–43. doi: 10.1038/nrc3184 22237392

[7] Kreso A Dick JE . Evolution of the cancer stem cell model. Cell Stem Cell (2014) 14(3):275–91. doi: 10.1016/j.stem.2014.02.006 24607403

[8] Ju F Atyah MM Horstmann N Gul S Vago R Bruns CJ . Characteristics of the cancer stem cell niche and therapeutic strategies. Stem Cell Res Ther (2022) 13(1):233. doi: 10.1186/s13287-022-02904-1 35659296 PMC9166529

[9] Plaks V Kong N Werb Z . The cancer stem cell niche: How essential is the niche in regulating stemness of tumor cells? Cell Stem Cell (2015) 16(3):225–38. doi: 10.1016/j.stem.2015.02.015 PMC4355577 25748930

[10] Hanahan D Coussens LM . Accessories to the crime: Functions of cells recruited to the tumor microenvironment. Cancer Cell (2012) 21(3):309–22. doi: 10.1016/j.ccr.2012.02.022 22439926

[11] Mare M Colarossi L Veschi V Turdo A Giuffrida D Memeo L . Cancer stem cell biomarkers predictive of radiotherapy response in rectal cancer: A systematic review. Genes-basel. (2021) 12(10):1502. doi: 10.3390/genes12101502 34680897 PMC8535834

[12] Calon A Lonardo E Berenguer-Llergo A Espinet E Hernando-Momblona X Iglesias M . Stromal gene expression defines poor-prognosis subtypes in colorectal cancer. Nat Genet (2015) 47(4):320–9. doi: 10.1038/ng.3225 25706628

[13] Qiu Y Wang L Zhong X Li L Chen F Xiao L . A multiple breast cancer stem cell model to predict recurrence of T1–3, N0 breast cancer. BMC Cancer (2019) 19(1):729. doi: 10.1186/s12885-019-5941-5 31340763 PMC6657050

[14] Lightner AL Chan T . Precision regenerative medicine. Stem Cell Res Ther (2021) 12(1):39. doi: 10.1186/s13287-020-02092-w 33413590 PMC7791634

[15] Zhao K Hu Y . Microbiome harbored within tumors: a new chance to revisit our understanding of cancer pathogenesis and treatment. Signal Transduct Target Ther (2020) 5(1):136. doi: 10.1038/s41392-020-00244-1 32728023 PMC7391753

[16] Nejman D Livyatan I Fuks G Gavert N Zwang Y Geller LT . The human tumor microbiome is composed of tumor type–specific intracellular bacteria. Science. (2020) 368(6494):973–80. doi: 10.1126/science.aay9189 PMC7757858 32467386

[17] Li Y Dong B Wu W Wang J Jin H Chen K . Metagenomic analyses reveal distinct gut microbiota signature for predicting the neoadjuvant chemotherapy responsiveness in breast cancer patients. Front Oncol (2022) 12:865121. doi: 10.3389/fonc.2022.865121 35433455 PMC9010823

[18] Siegel RL Miller KD Fuchs HE Jemal A . Cancer statistics, 2022. CA Cancer J Clin (2022) 72(1):7–33. doi: 10.3322/caac.21708 35020204

[19] Peschke K Jakubowsky H Schäfer A Maurer C Lange S Orben F . Identification of treatment-induced vulnerabilities in pancreatic cancer patients using functional model systems. EMBO Mol Med (2022) 14(4):e14876. doi: 10.15252/emmm.202114876 35119792 PMC8988213

[20] Bitzer M Ostermann L Horger M Biskup S Schulze M Ruhm K . Next-generation sequencing of advanced GI tumors reveals individual treatment options. Jco Precis Oncol (2020) 4(4). PO.19.00359. doi: 10.1200/PO.19.00359 PMC7446530 32923905

[21] Malinova A Veghini L Real FX Corbo V . Cell lineage infidelity in PDAC progression and therapy resistance. Front Cell Dev Biol (2021) 9:795251. doi: 10.3389/fcell.2021.795251 34926472 PMC8675127

[22] Thankamony AP Saxena K Murali R Jolly MK Nair R . Cancer stem cell plasticity – a deadly deal. Front Mol Biosci (2020) 7:79. doi: 10.3389/fmolb.2020.00079 32426371 PMC7203492

[23] Yang Y Meng WJ Wang ZQ . Cancer stem cells and the tumor microenvironment in gastric cancer. Front Oncol (2022) 11:803974. doi: 10.3389/fonc.2021.803974 35047411 PMC8761735

[24] Al-Hajj M Wicha MS Benito-Hernandez A Morrison SJ Clarke MF . Prospective identification of tumorigenic breast cancer cells. Proc Natl Acad Sci (2003) 100(7):3983–8. doi: 10.1073/pnas.0530291100 PMC153034 12629218

[25] Basu M Philipp LM Baines JF Sebens S . The microbiome tumor axis: How the microbiome could contribute to clonal heterogeneity and disease outcome in pancreatic cancer. Front Oncol (2021) 11:740606. doi: 10.3389/fonc.2021.740606 34631577 PMC8495218

[26] Valle S Martin-Hijano L Alcalá S Alonso-Nocelo M Sainz B . The ever-evolving concept of the cancer stem cell in pancreatic cancer. Cancers. (2018) 10(2):33. doi: 10.3390/cancers10020033 29373514 PMC5836065

[27] Hambardzumyan D Bergers G . Glioblastoma: Defining tumor niches. Trends Cancer (2015) 1(4):252–65. doi: 10.1016/j.trecan.2015.10.009 PMC4831073 27088132

[28] Lodestijn SC Miedema DM Lenos KJ Nijman LE Belt SC Makrini KE . Marker-free lineage tracing reveals an environment-instructed clonogenic hierarchy in pancreatic cancer. Cell Rep (2021) 37(3):109852. doi: 10.1016/j.celrep.2021.109852 34686335

[29] van Mackelenbergh MG Stroes CI Spijker R van Eijck CHJ Wilmink JW Bijlsma MF . Clinical trials targeting the stroma in pancreatic cancer: A systematic review and meta-analysis. Cancers. (2019) 11(5):588. doi: 10.3390/cancers11050588 31035512 PMC6562438

[30] Seino T Kawasaki S Shimokawa M Tamagawa H Toshimitsu K Fujii M . Human pancreatic tumor organoids reveal loss of stem cell niche factor dependence during disease progression. Cell Stem Cell (2018) 22(3):454–467.e6. doi: 10.1016/j.stem.2017.12.009 29337182

[31] Martínez-Bosch N Cristóbal H Iglesias M Gironella M Barranco L Visa L . Soluble AXL is a novel blood marker for early detection of pancreatic ductal adenocarcinoma and differential diagnosis from chronic pancreatitis. Ebiomedicine. (2021) 75:103797. doi: 10.1016/j.ebiom.2021.103797 34973624 PMC8724936

[32] Gui JC Yan WL Liu XD . CA19-9 and CA242 as tumor markers for the diagnosis of pancreatic cancer: a meta-analysis. Clin Exp Med (2014) 14(2):225–33. doi: 10.1007/s10238-013-0234-9 23456571

[33] Ammer-Herrmenau C Pfisterer N Weingarten MF Neesse A . The microbiome in pancreatic diseases: Recent advances and future perspectives. United Eur Gastroent (2020) 8(8):878–85. doi: 10.1177/2050640620944720 PMC7707879 32703080

[34] Geller LT Barzily-Rokni M Danino T Jonas OH Shental N Nejman D . Potential role of intratumor bacteria in mediating tumor resistance to the chemotherapeutic drug gemcitabine. Science (2017) 357(6356):1156–60. doi: 10.1126/science.aah5043 PMC5727343 28912244

[35] Kartal E Schmidt TSB Molina-Montes E Rodríguez-Perales S Wirbel J Maistrenko OM . A faecal microbiota signature with high specificity for pancreatic cancer. Gut (2022) 71:1359–72. doi: 10.1136/gutjnl-2021-324755 PMC9185815 35260444

[36] Rhim AD Mirek ET Aiello NM Maitra A Bailey JM McAllister F . EMT and dissemination precede pancreatic tumor formation. Cell (2012) 148(1–2):349–61. doi: 10.1016/j.cell.2011.11.025 PMC3266542 22265420

[37] Maruno T Fukuda A Goto N Tsuda M Ikuta K Hiramatsu Y . Visualization of stem cell activity in pancreatic cancer expansion by direct lineage tracing with live imaging. Elife (2021) 10:e55117. doi: 10.7554/eLife.55117 33393460 PMC7800378

[38] Westphalen CB Takemoto Y Tanaka T Macchini M Jiang Z Renz BW . Dclk1 defines quiescent pancreatic progenitors that promote injury-induced regeneration and tumorigenesis. Cell Stem Cell (2016) 18(4):441–55. doi: 10.1016/j.stem.2016.03.016 PMC4826481 27058937

[39] Zhu L Hissa B Győrffy B Jann JC Yang C Reissfelder C . Characterization of stem-like circulating tumor cells in pancreatic cancer. Diagnostics (2020) 10(5):305. doi: 10.3390/diagnostics10050305 32429174 PMC7278018

[40] Whatcott CJ Diep CH Jiang P Watanabe A LoBello J Sima C . Desmoplasia in primary tumors and metastatic lesions of pancreatic cancer. Clin Cancer Res (2015) 21(15):3561–8. doi: 10.1158/1078-0432.CCR-14-1051 PMC4526394 25695692

[41] Vonlaufen A Joshi S Qu C Phillips PA Xu Z Parker NR . Pancreatic stellate cells: Partners in crime with pancreatic cancer cells. Cancer Res (2008) 68(7):2085–93. doi: 10.1158/0008-5472.CAN-07-2477 18381413

[42] Provenzano PP Cuevas C Chang AE Goel VK Von Hoff DD Hingorani SR . Enzymatic targeting of the stroma ablates physical barriers to treatment of pancreatic ductal adenocarcinoma. Cancer Cell (2012) 21(3):418–29. doi: 10.1016/j.ccr.2012.01.007 PMC3371414 22439937

[43] Collisson EA Bailey P Chang DK Biankin AV . Molecular subtypes of pancreatic cancer. Nat Rev Gastroentero (2019) 16(4):207–20. doi: 10.1038/s41575-019-0109-y 30718832

[44] Moffitt RA Marayati R Flate EL Volmar KE Loeza SGH Hoadley KA . Virtual microdissection identifies distinct tumor- and stroma-specific subtypes of pancreatic ductal adenocarcinoma. Nat Genet (2015) 47(10):1168–78. doi: 10.1038/ng.3398 PMC4912058 26343385

[45] Initiative APCG Bailey P Chang DK Nones K Johns AL Patch AM . Genomic analyses identify molecular subtypes of pancreatic cancer. Nature. (2016) 531(7592):47–52. doi: 10.1038/nature16965 26909576

[46] Melendez-Zajgla J Maldonado V . The role of lncRNAs in the stem phenotype of pancreatic ductal adenocarcinoma. Int J Mol Sci (2021) 22(12):6374. doi: 10.3390/ijms22126374 34203589 PMC8232220

[47] Krieger TG Blanc SL Jabs J Ten FW Ishaque N Jechow K . Single-cell analysis of patient-derived PDAC organoids reveals cell state heterogeneity and a conserved developmental hierarchy. Nat Commun (2021) 12(1):5826. doi: 10.1038/s41467-021-26059-4 34611171 PMC8492851

[48] Nicolle R Gayet O Duconseil P Vanbrugghe C Roques J Bigonnet M . A transcriptomic signature to predict adjuvant gemcitabine sensitivity in pancreatic adenocarcinoma. Ann Oncol (2021) 32(2):250–60. doi: 10.1016/j.annonc.2020.10.601 33188873

[49] Bellotti R Speth C Adolph TE Lass-Flörl C Effenberger M Öfner D . Micro- and mycobiota dysbiosis in pancreatic ductal adenocarcinoma development. Cancers. (2021) 13(14):3431. doi: 10.3390/cancers13143431 34298645 PMC8303110

[50] Hosein AN Brekken RA Maitra A . Pancreatic cancer stroma: an update on therapeutic targeting strategies. Nat Rev Gastroentero (2020) 17(8):487–505. doi: 10.1038/s41575-020-0300-1 PMC8284850 32393771

[51] Tada K Ohta M Hidano S Watanabe K Hirashita T Oshima Y . Fucosyltransferase 8 plays a crucial role in the invasion and metastasis of pancreatic ductal adenocarcinoma. Surg Today (2020) 50(7):767–77. doi: 10.1007/s00595-019-01953-z 31950256

[52] Steinbichler TB Dudás J Skvortsov S Ganswindt U Riechelmann H Skvortsova II . Therapy resistance mediated by cancer stem cells. Semin Cancer Biol (2018) 53:156–67. doi: 10.1016/j.semcancer.2018.11.006 30471331

[53] Castillo ED Meier R Chung M Koestler DC Chen T Paster BJ . The microbiomes of pancreatic and duodenum tissue overlap and are highly subject specific but differ between pancreatic cancer and non-cancer subjects. Cancer Epidemiol Prev Biomarkers (2018) 28(2). doi: 10.1158/1055-9965.EPI-18-0542 PMC6363867 30373903

[54] Ambrosini G Pozza ED Fanelli G Carlo CD Vettori A Cannino G . Progressively de-differentiated pancreatic cancer cells shift from glycolysis to oxidative metabolism and gain a quiescent stem state. Cells (2020) 9(7):1572. doi: 10.3390/cells9071572 32605166 PMC7408749

[55] Bruschini S Ciliberto G Mancini R . The emerging role of cancer cell plasticity and cell-cycle quiescence in immune escape. Cell Death Dis (2020) 11(6):471. doi: 10.1038/s41419-020-2669-8 32555172 PMC7303167

[56] Riquelme E Zhang Y Zhang L Montiel M Zoltan M Dong W . Tumor microbiome diversity and composition influence pancreatic cancer outcomes. Cell. (2019) 178(4):795–806.e12. doi: 10.1016/j.cell.2019.07.008 31398337 PMC7288240

[57] Pushalkar S Hundeyin M Daley D Zambirinis CP Kurz E Mishra A . The pancreatic cancer microbiome promotes oncogenesis by induction of innate and adaptive immune suppression. Cancer Discov (2018) 8(4):403–16. doi: 10.1158/2159-8290.CD-17-1134 PMC6225783 29567829

[58] Frappart PO Hofmann TG . Pancreatic ductal adenocarcinoma (PDAC) organoids: The shining light at the end of the tunnel for drug response prediction and personalized medicine. Cancers (2020) 12(10):2750. doi: 10.3390/cancers12102750 32987786 PMC7598647

[59] Huch M Bonfanti P Boj SF Sato T Loomans CJM van de Wetering M . Unlimited *in vitro* expansion of adult bi-potent pancreas progenitors through the Lgr5/R-spondin axis. EMBO J (2013) 32(20):2708–21. doi: 10.1038/emboj.2013.204 PMC3801438 24045232

[60] Boj SF Hwang CI Baker LA Chio IIC Engle DD Corbo V . Organoid models of human and mouse ductal pancreatic cancer. Cell (2015) 160(1–2):324–38. doi: 10.1016/j.cell.2014.12.021 PMC4334572 25557080

[61] Breunig M Merkle J Melzer MK Heller S Seufferlein T Meier M . Differentiation of human pluripotent stem cells into pancreatic duct-like organoids. Star Protoc (2021) 2(4):100913. doi: 10.1016/j.xpro.2021.100913 34917972 PMC8669107

[62] Tiriac H Belleau P Engle DD Plenker D Deschênes A Somerville T . Organoid profiling identifies common responders to chemotherapy in pancreatic cancer. Cancer Discov (2018) 8(9):CD–18-0349. doi: 10.1158/2159-8290.CD-18-0349 PMC6125219 29853643

[63] Raghavan S Winter PS Navia AW Williams HL DenAdel A Lowder KE . Microenvironment drives cell state, plasticity, and drug response in pancreatic cancer. Cell (2021) 184(25):6119–6137.e26. doi: 10.1016/j.cell.2021.11.017 34890551 PMC8822455

[64] Driehuis E van Hoeck A Moore K Kolders S HE F MC G . Pancreatic cancer organoids recapitulate disease and allow personalized drug screening. Proc Natl Acad Sci (2019) 116(52):26580–90. doi: 10.1073/pnas.1911273116 PMC6936689 31818951

[65] Haque MR Wessel CR Leary DD Wang C Bhushan A Bishehsari F . Patient-derived pancreatic cancer-on-a-chip recapitulates the tumor microenvironment. Microsystems Nanoeng (2022) 8(1):36. doi: 10.1038/s41378-022-00370-6 PMC8971446 35450328

[66] de la Peña DO Trabulo SMD Collin E Liu Y Sharma S Tatari M . Bioengineered 3D models of human pancreatic cancer recapitulate *in vivo* tumour biology. Nat Commun (2021) 12(1):5623. doi: 10.1038/s41467-021-25921-9 34561461 PMC8463670

[67] Tsai S McOlash L Palen K Johnson B Duris C Yang Q . Development of primary human pancreatic cancer organoids, matched stromal and immune cells and 3D tumor microenvironment models. BMC Cancer (2018) 18(1):335. doi: 10.1186/s12885-018-4238-4 29587663 PMC5870823

[68] Schuth S Blanc SL Krieger TG Jabs J Schenk M Giese NA . Patient-specific modeling of stroma-mediated chemoresistance of pancreatic cancer using a three-dimensional organoid-fibroblast co-culture system. J Exp Clin Canc Res (2022) 41(1):312. doi: 10.1186/s13046-022-02519-7 PMC9588250 36273171

[69] Go YH Choi WH Bae WJ Jung SI Cho CH Lee SA . Modeling pancreatic cancer with patient-derived organoids integrating cancer-associated fibroblasts. Cancers. (2022) 14(9):2077. doi: 10.3390/cancers14092077 35565206 PMC9103557

[70] Cannone S Greco MR Carvalho TMA Guizouarn H Soriani O Molfetta DD . Cancer associated fibroblast (CAF) regulation of PDAC parenchymal (CPC) and CSC phenotypes is modulated by ECM composition. Cancers (2022) 14(15):3737. doi: 10.3390/cancers14153737 35954400 PMC9367491

[71] Chakladar J Kuo SZ Castaneda G Li WT Gnanasekar A Yu MA . The pancreatic microbiome is associated with carcinogenesis and worse prognosis in males and smokers. Cancers (2020) 12(9):2672. doi: 10.3390/cancers12092672 32962112 PMC7565819

[72] Thomas D Sagar S Liu X Lee HR Grunkemeyer JA Grandgenett PM . Isoforms of MUC16 activate oncogenic signaling through EGF receptors to enhance the progression of pancreatic cancer. Mol Ther (2021) 29(4):1557–71. doi: 10.1016/j.ymthe.2020.12.029 PMC8058431 33359791

[73] Guo W Zhang Y Guo S Mei Z Liao H Dong H . Tumor microbiome contributes to an aggressive phenotype in the basal-like subtype of pancreatic cancer. Commun Biol (2021) 4(1):1019. doi: 10.1038/s42003-021-02557-5 34465850 PMC8408135

[74] Pleguezuelos-Manzano C Puschhof J Huber AR van Hoeck A Wood HM Nomburg J . Mutational signature in colorectal cancer caused by genotoxic pks+E. coli Nature (2020) 580(7802):269–73. doi: 10.1038/s41586-020-2080-8 PMC8142898 32106218

[75] Puschhof J Pleguezuelos-Manzano C Martinez-Silgado A Akkerman N Saftien A Boot C . Intestinal organoid cocultures with microbes. Nat Protoc (2021) 16(10):4633–49. doi: 10.1038/s41596-021-00589-z 34381208

[76] Xing PY Pettersson S Kundu P . Microbial metabolites and intestinal stem cells tune intestinal homeostasis. Proteomics (2020) 20(5–6):1800419. doi: 10.1002/pmic.201800419 31994831

[77] Sodhi CP Neal MD Siggers R Sho S Ma C Branca MF . Intestinal epithelial toll-like receptor 4 regulates goblet cell development and is required for necrotizing enterocolitis in mice. Gastroenterology. (2012) 143(3):708–718.e5. doi: 10.1053/j.gastro.2012.05.053 22796522 PMC3584415

[78] Ferguson M Foley E . Microbial recognition regulates intestinal epithelial growth in homeostasis and disease. FEBS J (2022) 289(13):3666–91. doi: 10.1111/febs.15910 33977656

[79] Hou Y Wei W Guan X Liu Y Bian G He D . A diet-microbial metabolism feedforward loop modulates intestinal stem cell renewal in the stressed gut. Nat Commun (2021) 12(1):271. doi: 10.1038/s41467-020-20673-4 33431867 PMC7801547

[80] Shelkey E Oommen D Stirling ER Soto-Pantoja DR Cook KL Lu Y . Immuno-reactive cancer organoid model to assess effects of the microbiome on cancer immunotherapy. Sci Rep-uk (2022) 12(1):9983. doi: 10.1038/s41598-022-13930-7 PMC9200712 35705580

[81] Torrens-Mas M Perelló-Reus C Navas-Enamorado C Ibargüen-González L Sanchez-Polo A Segura-Sampedro JJ . Organoids: An emerging tool to study aging signature across human tissues. modeling aging with patient-derived organoids. Int J Mol Sci (2021) 22(19):10547. doi: 10.3390/ijms221910547 34638891 PMC8508868

[82] Lee Y Kang JS Ham OJ Son MY Lee MO . Gut metabolite trimethylamine n-oxide induces aging-associated phenotype of midbrain organoids for the induced pluripotent stem cell-based modeling of late-onset disease. Front Aging Neurosci (2022) 14:925227. doi: 10.3389/fnagi.2022.925227 36051303 PMC9426463

[83] Bauer M Nascakova Z Mihai AI Cheng PF Levesque MP Lampart S . The ALPK1/TIFA/NF-κB axis links a bacterial carcinogen to r-loop-induced replication stress. Nat Commun (2020) 11(1):5117. doi: 10.1038/s41467-020-18857-z 33037203 PMC7547021

[84] Hyun S Park D . Challenges in genomic analysis of model systems and primary tumors of pancreatic ductal adenocarcinoma. Comput Struct Biotechnol J (2022) 20:4806–15. doi: 10.1016/j.csbj.2022.08.064 PMC9464644 36147673

[85] Carlo CD Brandi J Cecconi D . Pancreatic cancer stem cells: Perspectives on potential therapeutic approaches of pancreatic ductal adenocarcinoma. World J Stem Cells (2018) 10(11):172–82. doi: 10.4252/wjsc.v10.i11.172 PMC6325076 30631392

[86] Zhang Z Feng H Qiu Y Xu Z Xie Q Ding W . Dysbiosis of gastric mucosal fungal microbiota in the gastric cancer microenvironment. J Immunol Res (2022) 2022:6011632. doi: 10.1155/2022/6011632 35340583 PMC8942701

[87] Shen W Tang D Wan P Peng Z Sun M Guo X . Identification of tissue-specific microbial profile of esophageal squamous cell carcinoma by full-length 16S rDNA sequencing. Appl Microbiol Biot (2022) 106(8):3215–29. doi: 10.1007/s00253-022-11921-2 35435458

[88] Liu K Zhao T Wang J Chen Y Zhang R Lan X . Etiology, cancer stem cells and potential diagnostic biomarkers for esophageal cancer. Cancer Lett (2019) 458:21–8. doi: 10.1016/j.canlet.2019.05.018 PMC6597177 31125642

[89] Wu Y Jiao N Zhu R Zhang Y Wu D Wang AJ . Identification of microbial markers across populations in early detection of colorectal cancer. Nat Commun (2021) 12(1):3063. doi: 10.1038/s41467-021-23265-y 34031391 PMC8144394

[90] Ren Z Li A Jiang J Zhou L Yu Z Lu H . Gut microbiome analysis as a tool towards targeted non-invasive biomarkers for early hepatocellular carcinoma. Gut (2019) 68(6):1014–23. doi: 10.1136/gutjnl-2017-315084 PMC6580753 30045880

